# Association between maternal and child minimum dietary diversity in urban informal settlements: evidence from a cross-sectional study in Mumbai, India

**DOI:** 10.1186/s40795-025-01201-3

**Published:** 2025-11-21

**Authors:** Melinda Mastan, Sheetal Rajan, Rijuta Sawant, Shanti Pantvaidya, Vanessa D’Souza, Sushmita Das

**Affiliations:** Society for Nutrition, Education and Health Action (SNEHA), Mumbai, India

**Keywords:** Dietary diversity, Maternal and child nutrition, Feeding behavior, Urban slums, India

## Abstract

**Background:**

Minimum dietary diversity (MDD) is associated with malnutrition, influencing children’s growth and development. Understanding the relationship between maternal and child MDD is crucial as mothers play a central role in food preparation and child feeding. This study examines the alignment between maternal and child MDD and the factors influencing each, to inform nutrition interventions in resource-limited settings.

**Methods:**

We analyzed cross-sectional data from 398 mother-child dyads (children aged 6–23 months) residing in urban informal settlements in Mumbai. Bivariate and multivariable logistic regression analyses were conducted to examine the association between maternal and child MDD and to identify factors associated with each.

**Results:**

Only 31.4% of children met MDD, compared to 43.5% of mothers. Maternal MDD was the strongest predictor of child MDD (AOR = 4.7, 95% CI: 2.813, 7.906). Child age and availing benefits of the Public Distribution System were also significantly associated with child MDD. For maternal MDD, their well-being and partner’s education level were key predictors. Despite shared household environments, children consumed fewer fruits, vegetables, and flesh foods than mothers, suggesting selective feeding practices.

**Conclusions:**

Behavior change communication should focus on promoting a shared meal between children and mothers. In addition, strengthening social protection schemes, supporting maternal well-being and engaging fathers in interventions may enhance dietary diversity outcomes for both mothers and children.

## Background

With fewer than five years left to achieve the Sustainable Development Goals (SDGs), malnutrition remains a major global challenge, contributing to nearly half of all deaths in children under five. As of 2022, an estimated 148 million children under five were stunted, 45 million were wasted, and 37 million were overweight worldwide [1]. Inadequate feeding practices directly cause malnutrition, affecting children’s growth, development, and overall health. Dietary diversity, a key indicator of feeding practices, is essential for ensuring diet quality, as no single food provides all the nutrients required for optimal physical and cognitive development [2, 3]. In recognition of its critical role, minimum dietary diversity (MDD) was formally included as a new SDG indicator in 2025, further underscoring the urgency of improving diet quality globally [4]. However, in India—home to the world’s largest child population—dietary diversity remains critically low, with only 23% of children aged 6–23 months meeting the MDD in 2021 [5]. In Maharashtra, this challenge is particularly acute, with over 80% of children aged 6–23 months lacking adequate dietary diversity according to recent studies [6].

Mumbai, the capital of Maharashtra, presents a paradoxical urban nutrition landscape. Despite greater access to food markets compared to rural areas, approximately 42% of the city’s 22 million residents live in informal settlements, many of whom are migrants from other regions of India [7]. This makes the city’s slum population highly diverse and uniquely vulnerable to nutritional challenges, particularly among young children. According to the National Family Health Survey (NFHS-5, 2019-21), 37.2% of children under five are stunted and 18.6% are wasted. Among children aged 6–23 months, only 17.8% receive an adequate diet [8]. These indicators challenge the notion of an ‘urban advantage’ in nutrition, revealing that urban poverty can create barriers to healthy diets as severe as those in rural contexts [9].

While child dietary diversity has been widely studied, there is growing evidence that maternal dietary diversity also plays a crucial role in shaping children’s diets. Studies in multiple low-middle income countries suggest that maternal and child dietary diversity are closely linked; children whose mothers consume a more diverse diet are significantly more likely to meet MDD [10, 11]. Yet, many existing interventions focus primarily on improving complementary feeding practices for children and separate these efforts from maternal diet interventions, limiting overall effectiveness [12]. Given that maternal food choices and access to diverse foods might influence what is available to children, assessing the alignment between maternal and child diets is important [13, 14]. In addition, identifying the key determinants of dietary diversity in both groups can inform more effective nutrition programs.

Existing research points to several socio-economic factors that influence maternal and child dietary diversity. Maternal education is a strong determinant, as higher education levels are associated with better dietary diversity in both mothers and children [10, 15, 16]. Household wealth also plays a significant role, with children from wealthier households more likely to meet MDD [10, 17]. Food insecurity remains a critical barrier, as mothers in low-resource settings often face limited access to diverse foods, negatively impacting both their own and their children’s diets [10, 18]. However, few studies have examined how psychosocial factors, such as maternal well-being, interact with socio-economic determinants to influence dietary diversity outcomes in vulnerable urban populations.

These nutritional issues are particularly challenging in urban slums such as those in Mumbai, where nearly half the city’s population resides [19]. These communities face unique risks due to rapid urbanization, overcrowding, and poor living conditions. Inadequate sanitation, limited access to nutrient-rich foods and constrained opportunities for household-level production further worsen nutritional vulnerabilities in these communities [20]. Despite the critical importance of understanding maternal and child dietary patterns in these settings, studies that concurrently assess maternal and child dietary diversity—while integrating household, psychosocial, and system-level determinants—remain limited. This gap is significant given the scale and complexity of nutritional challenges faced by mothers and children in Mumbai’s informal settlements, which differ markedly from those in rural areas and non-slum urban populations.

This study aims to address this gap by examining the relationship between maternal and child dietary diversity in urban informal settlements in Mumbai, India. It simultaneously assesses key determinants including maternal psychosocial well-being and access to social protection schemes. To our knowledge, this is among the first studies to adopt a comprehensive, integrated approach to dietary diversity in one of India’s most vulnerable urban populations. Findings from this study can inform the design of interventions that integrate maternal and child nutrition in resource-limited settings, ensuring that interventions address both direct and underlying determinants of dietary diversity in urban poor populations.

## Methods

### Setting

The M East Ward of Mumbai is widely recognized as one of the city’s most socio-economically marginalized administrative divisions, with recent assessments indicating that approximately 77–80% of its residents live in informal settlements. Characterized by high population density and widespread informal housing, the ward faces persistent deficits in basic public services, including access to piped drinking water, improved sanitation facilities and reliable waste management. These gaps, coupled with insecure housing tenure and limited livelihood opportunities, contribute to chronic public health challenges and compound vulnerabilities among its largely migrant population [21, 22]. This study was conducted in two large informal settlements located in Mankhurd within the M East Ward.

### Study design

This study is based on a cross-sectional survey conducted in April-May 2024, following the implementation of a community-based intervention implemented by the Society for Nutrition, Education and Health Action (SNEHA), a Mumbai-based non-governmental organization focused on maternal and child health and nutrition. SNEHA’s established presence in these informal settlements facilitated community engagement and enabled stratified random sampling, with Anganwadi catchment areas serving as strata. Anganwadis are government-supported childcare centers under India’s Integrated Child Development Services (ICDS) scheme, providing health and nutrition services to mothers and young children [23]. This paper presents a secondary cross-sectional analysis focusing on maternal and child dietary diversity and its key determinants.

### Sample

The survey included a total of 847 households, of which 398 participants had at least one child aged 6–23 months. These 398 mother-child dyads constitute the study population for this analysis. Mothers were eligible to participate if they had resided in the intervention area for at least six months and provided informed consent. The six-month minimum residency criterion was chosen to ensure that participants had sufficient exposure to local maternal and child health services, including those delivered through SNEHA and government programs such as ICDS. Given that in and out migration is frequent in informal settlements, this residency threshold provides a balance between capturing stable service use and maximizing sample inclusiveness. Households were excluded if the mother was unavailable after three follow-up visits or if they did not meet the minimum residency duration requirement.

### Data collection

The survey employed a stratified random sampling method, with Anganwadi center catchment areas serving as the strata. Within each stratum, a random sample of households with children aged 0–5 years was drawn from a pre-existing line list of eligible households. A team of 12 interviewers, supervised by two field officers, was responsible for data collection.

Data were gathered through face-to-face interviews with mothers, using a structured questionnaire capturing information on maternal and child dietary diversity, socio-demographic factors, maternal well-being, male engagement and access to social protection schemes. The questionnaire incorporated standardized tools, including the World Health Organization (WHO) & United Nations Children’s Fund (UNICEF) 24-hour dietary recall for child MDD [24], the Food and Agriculture Organization (FAO) MDD-W 24-hour recall for maternal dietary diversity [25], the Short Warwick-Edinburgh Mental Well-being Scale (SWEMWBS) [26], and socio-demographic questions adapted from the NFHS-5 [5].

While the dietary diversity tools have been widely applied in national surveys across India, SWEMWBS is a validated psychometric scale that has been used in select studies within the Indian context. Where available, existing Hindi versions of the tools were used. Otherwise, items were translated from English to Hindi and back-translated by two independent translators, with an emphasis on preserving contextual meaning rather than literal translation. The translated tools were pilot-tested and subsequently refined.

Data was collected using CommCare (Dimagi, USA), an open-source mobile-based platform that ensures accurate data entry and secure storage on a cloud-based server.

### Key outcome variables

#### Child MDD

The primary outcome variable, child minimum dietary diversity, was assessed using a 24-hour dietary recall based on maternal reports of the child’s food intake. Data were collected using the WHO-recommended questionnaire, where mothers were asked to recall all foods consumed by their child in the past 24 hours. Reported foods were categorized into 8 food groups: (1) breastmilk; (2) grains; (3) legumes and nuts; (4) dairy products; (5) flesh foods (meat, fish, poultry); (6) eggs; (7) vitamin-A rich fruits and vegetables; (8) other fruits and vegetables. Each food group was coded as either “consumed” (score 1) or “not consumed” (score 0). A child was considered to have met MDD if they consumed food from ≥ 4 food groups in the past 24 hours.

#### Maternal MDD

The maternal MDD was also assessed using 24-hour dietary recall, following the Minimum Dietary Diversity for Women (MDD-W) indicator by FAO. Maternal MDD-W is categorized as yes if consuming > 5 food groups from the ten food groups: (1) grain, white, sugary; (2) pulse; (3) nuts; (4) dairy; (5) organ, poultry, meat, fish, insect; (6) egg; (7) green leaves vegetables; (8) orange and red fruit; (9) other fruits; (10) other vegetables.

### Covariates

Child characteristics included age and sex. Age was categorized into two groups: 6–11 months and 12–23 months. Sex was classified as male or female. Maternal characteristics included age, education, occupation, antenatal care (ANC) registration, well-being, and male engagement. Maternal age was grouped into four categories: less than 25 years, 25–29 years, 30–34 years, and 35 years or older. Maternal education was categorized as never attended school, primary (1–5 years of schooling), secondary (6–10 years), and higher (11–17 years). Occupation was classified as working or not working. Timing of ANC registration was defined as the trimester in which the woman first accessed care, categorized into first trimester (within the first three months) or second trimester onwards (four months or later).

Maternal well-being was assessed using the SWEMWBS, a 7-item tool scored on a range of 7 to 35, where higher scores indicate greater well-being [26]. Based on the scores, well-being was categorized as low (7–15.9), medium (16–25.9), or high (≥ 26). Male engagement was assessed through a binary variable, indicating whether the husband had helped with household chores in the past week (yes/no).

Paternal characteristics included education and occupation. Paternal education was categorized similarly to maternal education. Paternal occupation was categorized into three categories: not working, working in the informal sector, and working in the formal sector. Informal sector occupations included roles that typically do not require formal skills or training such as manufacturing and factory work. Formal sector occupations included professional, technical, or other skilled service roles.

Household characteristics encompassed socioeconomic status (SES), parity, household size, household type, and receipt of social protection benefits. SES was assessed using a principal component analysis of variables such as ownership of house and ration card, housing quality (e.g., roof materials), and household assets [27]. Parity was categorized into 1–2 children or three or more children. Household size reflected the total number of members, while household type was categorized as nuclear or joint family. Receipt of benefits from the Public Distribution System (a social protection scheme in India providing subsidized food and non-food items to the poor through a network of Fair Price Shops or Ration Shops, ensuring food security), was assessed for the past year (yes/no) [28].

### Statistical analysis

Data analysis was conducted using Stata 18.0. Descriptive statistics were used to summarize the demographic and household characteristics of the study population, including child, maternal, paternal, and household variables. Bivariate analysis was performed using Chi-square tests to examine associations between each covariate and child MDD.

A logistic regression analysis was conducted to assess the relationship between maternal and child MDD, as well as the factors influencing child MDD and maternal MDD-W. The regression models were adjusted for covariates using a three-step approach:

- Model 1: Examined the association between child MDD and maternal and child characteristics.
- Model 2: Added socio-demographic factors, including paternal and household characteristics.
- Model 3: Included maternal MDD, adjusting for all variables in Models 1 and 2.

Multicollinearity was assessed using Variance Inflation Factors (VIF), with a mean VIF of 2.69 indicating acceptable collinearity. While most variables had VIF values well below 3, paternal occupation exhibited a high VIF (> 15). However, it was retained due to its theoretical relevance. Sensitivity analyses excluding this variable did not alter the primary association.

Model calibration was evaluated using the Hosmer-Lemeshow goodness-of-fit test (χ² = 7.76, df = 8, *p* = 0.458), indicating adequate fit. Internal consistency of the SWEMWBS was assessed using Cronbach’s alpha (α = 0.615), which is considered acceptable for brief scales.

In all analyses, statistical significance was set at *p*-value < 0.05, and odds ratios along with their 95% confidence intervals were used to assess the strength of association.

### Ethical considerations

The study protocol was reviewed and approved by the Institutional Review Board of Sigma Research & Consulting Pvt Ltd., New Delhi (10076/IRB/23–24). This review board was selected for its recognized expertise in ethical oversight of research in India, including in Mumbai. The study was conducted in accordance with the Declaration of Helsinki. Interviewers respected the privacy and confidentiality of all participants and sought written informed consent prior to interview. Confidentiality of the information was maintained in data processing and outputs did not include participants’ names/identifiers.

## Results

### Demographic characteristics

Among the 398 children aged 6–23 months included in this study, 64.3% were between 12 and 23 months old. The gender distribution was nearly equal, with 50.7% male and 49.2% female. Mothers were primarily aged 25–29 years (39.9%), while 35.6% were under 25 years old. Nearly two-fifths had attained secondary education (38.1%), most identified as Muslim (73.1%), and were not employed (87.6%). More than half (58.2%) attended their ANC visit during the first trimester. Additionally, 52.2% reported that their husbands did not assist with household chores in the past week. The majority of mothers had a moderate level of well-being (71.8%). Socioeconomic status was fairly distributed, with 28.6% classified as least poor, 23.6% in quartile 3, 22.1% in quartile 2, and 25.6% in the poorest category. Most households had fewer than three children (64.5%) and 67.8% had more than five members living in the same house. Nuclear families accounted for 56.2% of households. Regarding fathers, most had completed secondary education (80.6%) and worked in the informal sector (81.9%). 20% of families had received benefits of the Public Distribution System (PDS). Table 1 shows participants’ characteristics.

**Table 1:** Child, maternal, and household characteristics of the study participants (*N* = 398)

Characteristics	*n*	%
*Child Characteristics*		
Age (months)		
6–11	142	35.6%
12–23	256	64.3%
Sex		
Male	202	50.7%
Female	196	49.2%
*Maternal Characteristics*		
Age (years)		
< 25	142	35.6%
25–29	159	39.9%
30–34	67	16.8%
≥ 35	30	7.5%
Education		
No schooling	84	21.1%
Primary	67	16.8%
Secondary	152	38.1%
Higher	95	23.8%
Religion		
Muslim	291	73.1%
Hindu	107	26.8%
Occupation		
Not working	349	87.6%
Working	49	12.3%
Timing of ANC registration		
1 st trimester	229	58.2%
≥ 2nd trimester	164	41.7%
Male Engagement		
No	208	52.2%
Yes	185	46.4%
* Missing*	*5*	*1.2%*
Maternal wellbeing		
Low wellbeing	88	22.1%
Moderate wellbeing	286	71.8%
High wellbeing	24	6.0%
*Paternal Characteristics*		
Father’s education		
No schooling	73	18.8%
Primary	60	34.2%
Secondary	180	80.6%
Higher	75	19.3%
Father’s occupation		
Not working	4	1.0%
Informal	322	81.9%
Formal	67	17.0%
*Household Characteristics*		
Socio-economic status		
Poorest	102	25.6%
Quartile 2	88	22.1%
Quartile 3	94	23.6%
Least poor	114	28.6%
Parity		
1–2 children	257	64.5%
3 or more children	141	35.4%
Household size		
≥ 5	270	67.8%
< 5	128	32.1%
Household type		
Nuclear	224	56.2%
Joint	174	43.7%
Received benefits of Public Distribution System		
No	317	79.6%
Yes	81	20.3%

### Maternal and child minimum dietary diversity

In this study, only 31.4% of children met the MDD criteria, while a slightly higher percentage of mothers (43.5%) achieved MDD.

**Fig. 1:**
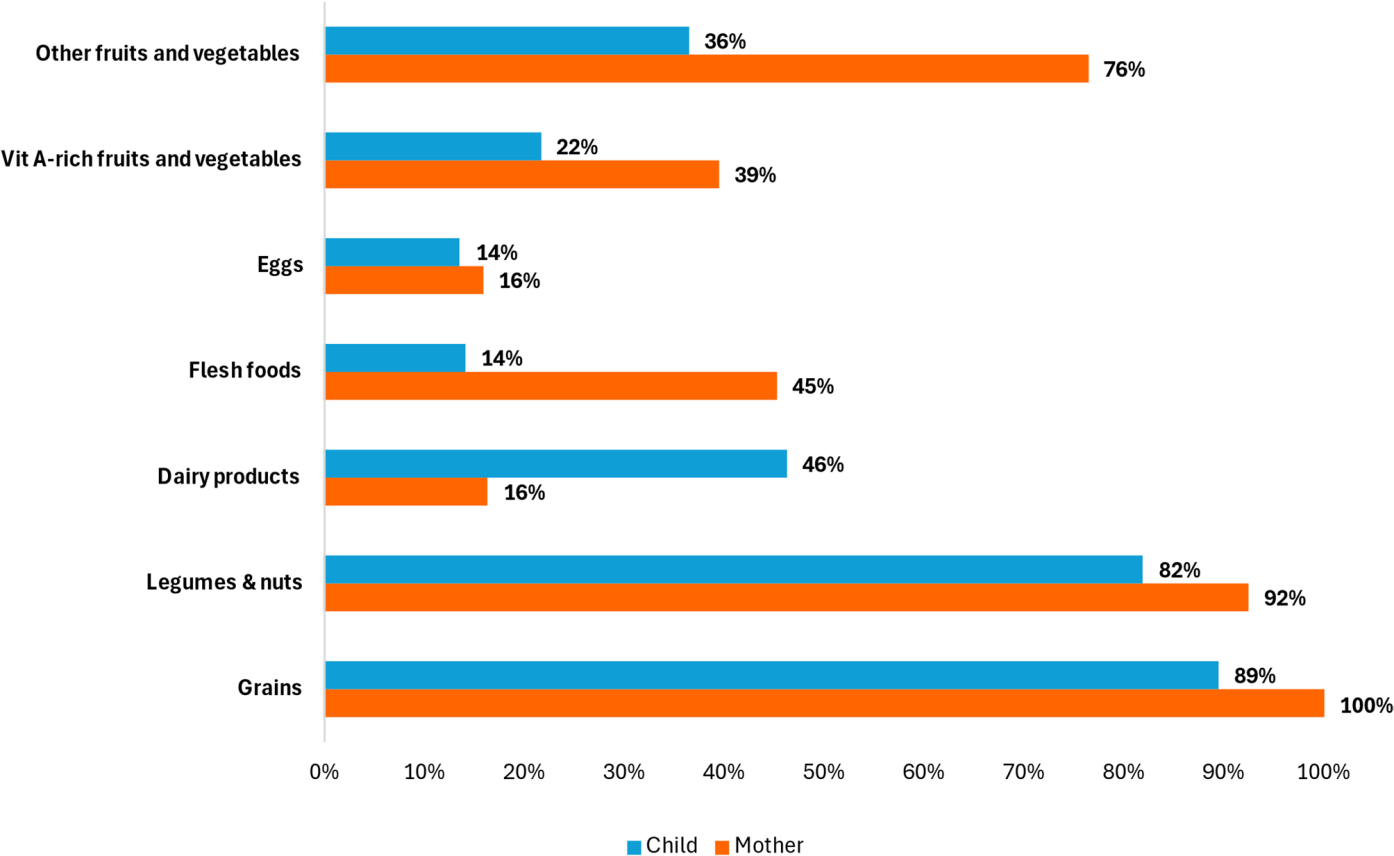
Dietary Diversity Patterns of Mothers and Children in the Study Population (*N* = 398)

To facilitate a direct visual comparison of dietary patterns between mothers and children, we regrouped food groups and excluded breastmilk from the child MDD exclusively for descriptive analysis, as shown in Fig. 1. Maternal MDD-W food groups were also regrouped into seven categories to align with child MDD categories. This regrouping excluded breastmilk from child MDD, and combined pulses and nuts; vitamin A-rich fruits and vegetables; and other fruits and vegetables.

When comparing the food group consumption by mothers and children (Fig. 1), notable differences emerged: eggs, legumes, and grains were consumed almost equally, but mothers consumed more flesh foods and fruits and vegetables, whereas children consumed more dairy. Fruits, vegetables, and flesh foods showed the lowest overlap, as children consumed these items significantly less often.

### Determinants of child MDD

Bivariate analyses showed a significant association between maternal MDD and child MDD (*p* < 0.0001). Other variables that were positively associated with child MDD included child age and receiving PDS benefits. Details are presented in Table 2.

### Association between maternal and child MDD

We constructed three models to examine factors associated with child MDD. In the first model, we explored the relationship between child MDD and child and maternal characteristics. We found that only child age was a significant predictor of child MDD (*p* < 0.001), with older children having 2.6 times higher odds of achieving MDD than younger children (6–11 months old) (AOR = 2.6, 95% CI: 1.6, 4.4).

In the second model, we extended the analysis by including household characteristics. Child age remained a significant predictor, and receiving PDS benefits also emerged as a predictor. Children in households benefiting from social protection in the form of subsidized food/non-food items were 2.5 times more likely to achieve MDD (AOR = 2.5, 95% CI: 1.4, 4.5).

In the final model, we incorporated maternal MDD. Child age, PDS benefits, and maternal MDD were all significant predictors of child MDD. Maternal MDD was the strongest predictor, as children of mothers who met the MDD criteria were 4.7 times more likely to achieve MDD (AOR = 4.7, 95% CI: 2.8, 7.9). Notably, the odds of achieving MDD associated with child age and PDS access increased after accounting for maternal MDD, suggesting that maternal dietary diversity may strengthen the relationship between these factors and child MDD. Details are presented in Table 2. 

**Table 2:** Association of child MDD with maternal MDD and factors including child, maternal, paternal and household characteristics

Variables	Bivariate		Model 1		Model 2		Model 3	
	OR	95% CI	AOR	95% CI	AOR	95% CI	AOR	95% CI
Maternal MDD								
No							1	[1,1]
Yes	4.018***	[2.565, 6.294]					4.716***	[2.813,7.906]
*Child Characteristics*								
Age								
6–11 months			1	[1,1]	1	[1,1]	1	[1,1]
12–23 months	2.642***	[1.620, 4.308]	2.605***	[1.554, 4.368]	2.712***	[1.570, 4.685]	3.007***	[1.686, 5.364]
Sex								
Male			1	[1,1]	1	[1,1]	1	[1,1]
Female	1.485	[0.970, 2.273]	1.414	[0.899, 2.222]	1.535	[0.954, 2.470]	1.531	[0.926, 2.531]
*Maternal Characteristics*								
Age (years)								
< 25			1	[1,1]	1	[1,1]	1	[1,1]
25–29	1.092	[0.668, 1.786]	0.903	[0.531, 1.534]	0.869	[0.488,1.549]	0.839	[0.455,1.546]
30–34	1.417	[0.768, 2.615]	1.201	[0.615, 2.347]	0.876	[0.378, 2.028]	0.787	[0.317,1.956]
35+	0.866	[0.357, 2.100]	0.82	[0.315, 2.137]	0.693	[0.222, 2.164]	0.648	[0.195, 2.153]
Education								
No schooling			1	[1,1]	1	[1,1]	1	[1,1]
Primary	1.234	[0.620, 2.454]	1.13	[0.544, 2.346]	1.064	[0.485, 2.335]	0.965	[0.422, 2.207]
Secondary	0.961	[0.536, 1.725]	1.095	[0.572, 2.093]	1.064	[0.521, 2.174]	0.841	[0.391,1.809]
Higher	1.256	[0.669, 2.359]	1.442	[0.694, 2.998]	1.064	[0.459, 2.469]	1.004	[0.409, 2.466]
Religion								
Muslim			1	[1,1]	1	[1,1]	1	[1,1]
Hindu	0.804	[0.494, 1.309]	0.733	[0.416, 1.291]	0.708	[0.381, 1.319]	0.82	[0.429,1.566]
Occupation								
Not working			1	[1,1]	1	[1,1]	1	[1,1]
Working	0.677	[0.340, 1.349]	0.56	[0.265,1.186]	0.465	[0.206,1.049]	0.57	[0.238, 1.367]
Timing of ANC registration								
1 st trimester			1	[1,1]	1	[1,1]	1	[1,1]
2nd/3rd trimester	0.956	[0.620, 1.476]	1.036	[0.650,1.652]	1.001	[0.606,1.654]	0.957	[0.559, 1.636]
Male engagement								
No			1	[1,1]	1	[1,1]	1	[1,1]
Yes	0.875	[0.571, 1.339]	1.01	[0.630, 1.619]	1.033	[0.620, 1.722]	0.936	[0.546, 1.607]
Wellbeing								
Low wellbeing			1	[1,1]	1	[1,1]	1	[1,1]
Moderate wellbeing	1.540	[0.896, 2.645]	1.574	[0.889, 2.786]	1.546	[0.848, 2.818]	1.339	[0.708, 2.533]
High wellbeing	1	[0.353, 2.836]	0.914	[0.305, 2.733]	0.834	[0.263, 2.645]	0.535	[0.155, 1.846]
*Paternal Characteristics*								
Father’s education								
No schooling					1	[1,1]	1	[1,1]
Primary	1.218	[0.569, 2.606]			1.037	[0.440, 2.445]	0.799	[0.324,1.967]
Secondary	1.317	[0.716, 2.424]			1.451	[0.715, 2.944]	1.128	[0.534, 2.384]
Higher	1.693	[0.839, 3.415]			1.995	[0.810, 4.916]	1.53	[0.587, 3.991]
Father’s occupation								
Not working					1	[1,1]	1	[1,1]
Informal	1.293	[0.133, 12.590]			1.761	[0.167,18.56]	1.693	[0.154,18.66]
Formal	2.025	[0.200, 20.508]			3.051	[0.272, 34.17]	3.497	[0.298, 41.08]
*Households Characteristics*								
SES								
Poorest					1	[1,1]	1	[1,1]
Quartile 2	0.588	[0.308, 1.123]			0.531	[0.256, 1.100]	0.544	[0.253, 1.170]
Quartile 3	1.032	[0.571, 1.868]			0.871	[0.427, 1.776]	0.857	[0.397, 1.849]
Least poor	1.040	[0.591, 1.830]			0.755	[0.356, 1.602]	0.715	[0.320, 1.599]
Parity								
1–2 children					1	[1,1]	1	[1,1]
3 or more children	0.872	[0.561, 1.353]			1.664	[0.747, 3.704]	1.777	[0.761, 4.146]
Household size								
>=5					1	[1,1]	1	[1,1]
< 5	1.160	[0.740, 1.818]			1.391	[0.644, 3.001]	1.596	[0.708, 3.593]
Household type								
Nuclear					1	[1,1]	1	[1,1]
Joint	1.248	[0.812, 1.919]			0.857	[0.440, 1.666]	0.987	[0.490, 1.989]
Received benefits of Public Distribution System								
No					1	[1,1]	1	[1,1]
Yes	2.049***	[1.240, 3.386]			2.496**	[1.374, 4.534]	2.577**	[1.365, 4.865]

**p< 0.01, ***p< 0.001

### Factors associated with maternal MDD

**Fig. 2:**
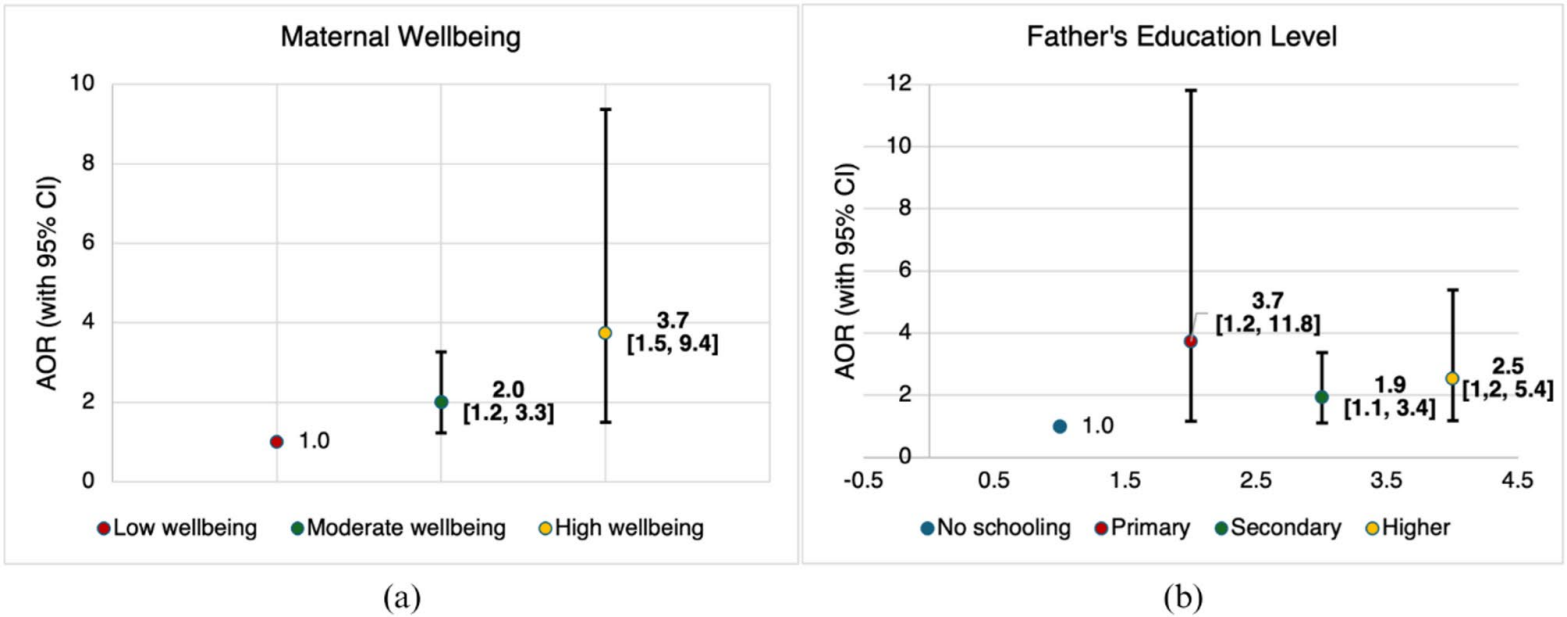
Factors associated with maternal MDD. **a** Panel (**a**) shows the association between levels of maternal wellbeing and odds of achieving maternal MDD, with low wellbeing as the reference category. **b** Panel (**b**) shows the association between paternal education level and maternal MDD, using no schooling as the reference. Error bars indicate 95% confidence intervals

We also ran a logistic regression analysis to find factors associated with maternal MDD from the maternal, paternal and household characteristics (Fig. 2). Mothers with moderate well-being had 2 times higher odds of achieving MDD compared to those with low well-being (AOR = 2.0, 95% CI: 1.2–3.3, *p* < 0.01). Similarly, mothers with high well-being were 3.7 times more likely to achieve MDD (AOR = 3.7, 95% CI: 1.5–9.4, *p* < 0.01).

The education level of the father was also a significant predictor of maternal MDD. Mothers whose husbands had primary education had 3.7 times higher odds of achieving MDD compared to those whose husbands had no schooling (AOR = 3.7, 95% CI: 1.2–11.8, *p* < 0.05). Additionally, mothers with husbands who attained secondary education had 1.9 times higher odds of achieving MDD (AOR = 1.9, 95% CI: 1.1–3.4, *p* < 0.05), while those whose husbands had higher education had 2.5 times higher odds (AOR = 2.5, 95% CI: 1.2–5.4, *p* < 0.05).

Other factors, including maternal age, occupation, household SES and household composition, were not significantly associated with maternal MDD.

## Discussion

This study explored maternal and child MDD and their relationship in urban informal settlements of Mumbai, India. Only 31.4% of children aged 6–23 months achieved MDD, which is fewer compared to 43.5% of mothers. Maternal MDD emerged as the strongest predictor of child MDD. Children with mothers who achieved MDD are more likely to achieve MDD as well. This finding is consistent with previous studies from urban slums in Delhi, as well as in Nigeria, Southern Ethiopia, and Lebanon, where similar associations were observed [29–32]. This may be explained by the fact that mothers are primarily responsible for preparing meals for the household, and their food choices influence both the child’s diet and the availability of foods at home [18, 33].

However, there is a gap in food group consumption between mothers and children. While both commonly consumed grains, legumes, and nuts, children were less likely to consume flesh foods, fruits, and vegetables. Egg consumption, in particular, was low among both mothers and children. Dairy products were the only food group more commonly consumed by children than mothers. These discrepancies suggest selective feeding practices that may stem from perceptions regarding which food groups are appropriate for young children [11, 30]. These include concerns that vegetables may be too hard in texture and may cause choking, or that eggs may cause indigestion and are therefore unsuitable for this age group [34, 35].

Moreover, low maternal self-efficacy and intra-household food sharing may also contribute to the observed discrepancies. A qualitative study in Mumbai found that mothers were hesitant to introduce new foods or recipes due to fears that the child might reject them, fall ill, or that family members would criticize their choices [34]. Additionally, even when eggs are distributed through food assistance programs in India, they are often shared among household members resulting in limited intake by the intended child. Intra-household food allocation practices often place young children at disadvantage, where they are frequently served last or receive smaller portions compared to the adults [35, 36].

The selective allocation of foods in children’s diets, despite their presence in maternal diets, reflects a disconnect between household food access and child feeding practices. Rather than a lack of access, this points to missed opportunities within the home. In particular, children consumed fewer animal protein and micronutrient-rich foods, which are essential for growth and development during the critical first 1000 days of their lives [37]. Addressing this gap requires shifting maternal perceptions, promoting shared family meals, and introducing children to the family pot at six months of age while ensuring appropriate food textures based on their developmental stage to support diverse and nutrient-rich intake [11].

Furthermore, child age was significantly associated with child MDD, with older children (12–23 months) more likely to meet MDD compared to younger ones. This trend is observed in other studies from India [38, 39], and globally [40]. This is plausible, as older children relatively eat more frequently and in larger quantities, increasing their exposure to a wider range of food groups. Caregivers may also perceive certain foods as more appropriate for older children, and the current complementary feeding counselling often promotes stepwise food introduction. This message may be interpreted conservatively by caregivers, resulting in delayed inclusion of diverse foods during early complementary feeding [38, 40, 41]. Therefore, counselling practices need to ensure that caregivers clearly understand how to appropriately introduce diverse foods from six months of age. Investment should focus on strengthening collaborative efforts among frontline healthcare workers—such as Anganwadi workers, Accredited Social Health Activists and Auxiliary Nurse Midwives—in enhancing the quality and frequency of contact with caregivers. This can be achieved through capacity building and behavior change communication to improve both maternal and child dietary diversity, as demonstrated by evidence from Maharashtra [42].

In addition to child age, access to subsidized food and non-food items under India’s PDS, was also associated with child MDD. The PDS is a national social protection scheme that provides essential food items such as grains, legumes, sugar, and oil to eligible households [43]. Many families depend on this program for food, which explains the high consumption of grains and legumes in our study. By covering basic staples, families can allocate their resources to buy other food groups, increasing their chances of meeting MDD. This finding aligns with previous research that nutrition-sensitive social protection schemes, such as cash transfers and food subsidies help improve dietary diversity in India [44]. Social protection programs are especially important in urban slums, where limited space and resources make home gardening or agricultural interventions challenging [17]. However, access to PDS benefits depends on possession of valid identification documents, such as ration cards, which are often required for enrollment. Although approximately half of the households in our sample reported holding ration cards, many families, particularly migrants and recent arrivals in informal settlements, may face significant barriers due to lack of documentation. These persistent challenges of limited access and concerns over food quality may limit families from fully benefiting from the PDS program [43]. Addressing these gaps is required to improve dietary diversity and nutrition among vulnerable households.

Our study also found several factors that were uniquely associated with maternal MDD. Maternal well-being and the father’s education level emerged as key predictors. Mothers with moderate to high well-being scores are more likely to achieve MDD compared to those with low well-being scores. Well-being in this context includes thinking clearly and being able to make their own mind about things, which may enable mothers to pay greater attention to food preparation and self-care. This finding aligns with existing literature showing a positive association between maternal mental health and their dietary diversity, and also when mothers have greater autonomy, particularly in food purchasing decisions, they have a higher chance of meeting MDD [16, 45].

Fathers’ education was significantly associated with maternal MDD, similar to previous studies [46–48]. Higher educational attainment among male partners may increase awareness of nutritional needs and encourage more diverse food choices within the household [47, 48]. Supportive attitudes and behaviors from fathers have also been linked to higher child dietary diversity, reinforcing the role of household dynamics in shaping nutrition practices [17].

This research offers important evidence on maternal and child dietary diversity in urban informal settlements, which adds to the sparse literature on dietary habits in vulnerable populations in India. While previous research has demonstrated associations between maternal and child dietary diversity, there remains a paucity of studies examining these dynamics in India’s urban informal settlements, where unique challenges, such as high migration and resource scarcity, intersect. This study addresses the gap by analyzing maternal and child MDD concurrently and highlighting the influence of maternal psychosocial health, paternal education and social protection access within these vulnerable populations. The integration of these factors not only advances understanding beyond what is typically expected but also identifies neglected leverage points for intervention, such as supporting maternal mental health and reducing structural barriers for marginalized urban families. However, it is not without limitations. First, the relatively small sample size (*N* = 398 mother-child dyads), drawn as a subset of children aged 6–23 months from a larger survey, limits the generalizability of the findings. Second, the cross-sectional design limits causal interpretations of the observed associations between maternal and child MDD, and other determinants, including access to social protection schemes. Third, while the study highlights dietary diversity patterns, it does not directly assess the relationship between maternal dietary practices and child nutritional status indicators, such as stunting, wasting or underweight, which would have strengthened the conclusions. Other limitations include potential recall bias in dietary data collection and the lack of qualitative exploration into cultural or behavioral factors influencing selective food allocation to children.

Despite these limitations, this study makes significant contributions to understanding dietary practices in urban informal settlements. It identifies maternal MDD as a key determinant of child MDD, with maternal well-being emerging as an important predictor of maternal dietary diversity. This underscores the need to address maternal psychosocial health alongside dietary interventions. By bringing attention to food group consumption patterns and the influence of social protection schemes such as the PDS, the study offers actionable insights for enhancing dietary diversity. These results provide a basis for informing interventions and policies to improve nutrition in vulnerable urban populations. Specifically, policies that enhance the coverage and inclusivity of social protection schemes like the PDS should be strengthened to ensure equitable access for migrant households. Additionally, nutrition programs should prioritize integrated maternal and child dietary diversity interventions, incorporating maternal psychosocial health support. Training programs for frontline workers should be expanded with a focus on behavior change communication, counseling skills and maternal mental health awareness. Empowering these workers to deliver culturally sensitive, context-specific guidance can improve caregiver knowledge and practice around early and appropriate complementary feeding. Future research should consider longitudinal designs to establish causal relationships and incorporate qualitative methods to explore cultural and behavioral determinants of dietary practices further.

## Conclusions

Child MDD is strongly associated with maternal MDD, with children more likely to meet MDD when their mothers do. However, differences in the specific food groups consumed suggest the presence of missed opportunities within households rather than limited access. Child MDD is also influenced by factors such as age and access to PDS, while maternal MDD is influenced by maternal well-being and the father’s education levels. To improve dietary diversity among children, interventions should focus on aligning maternal and child diets, promoting shared family meals, and implementing practical strategies such as enhancing frontline worker capacity, scaling up behavior change communication, improving accessibility and inclusivity of social protection schemes for migrant populations and integrating maternal psychosocial support. Engaging fathers and empowering caregivers with culturally appropriate counseling are essential steps for sustainable change. Strengthening these factors could contribute to better maternal and child dietary diversity.

## Data Availability

The dataset supporting the conclusions of this article is available in the Open Science Framework repository, https://osf.io/rudnj/.

## Abbreviations

ANC: Antenatal care
AOR: Adjusted odds ratio
CI: Confidence interval
FAO: Food and Agriculture Organization
ICDS: Integrated Child Development Services
MDD: Minimum Dietary Diversity
MDD-W: Minimum Dietary Diversity for Women
NFHS: National Family Health Survey
PDS: Public Distribution System
SWEMWBS: Short Warwick-Edinburgh Mental Wellbeing Scale
SDG: Sustainable Development Goals
SES: Socioeconomic status
SNEHA: Society for Nutrition, Education and Health Action
UNICEF: United Nation’s Children Fund
VIF: Variance Inflation Factor
WHO: World Health Organization
